# Strategy for Encapsulation of CdS Quantum Dots into Zeolitic Imidazole Frameworks for Photocatalytic Activity

**DOI:** 10.3390/nano10122498

**Published:** 2020-12-12

**Authors:** Ye Rim Son, Minseok Kwak, Songyi Lee, Hyun Sung Kim

**Affiliations:** 1 Department of Chemistry, Pukyong National University, Busan 48513, Korea; syl75218@daum.net (Y.R.S.); mkwak@pukyong.ac.kr (M.K.); 2 Department of Industry 4.0 Convergence Bionics Engineering, Pukyong National University, Yongso-ro, Nam-gu, Busan 48513, Korea

**Keywords:** quantum dot, cadmium sulfide, zeolitic imidazole frameworks, photocatalyst

## Abstract

Encapsulating CdS quantum dots (QDs) into zeolitic imidazole framework-8 (ZIF-8) can offer several advantages for photocatalysis. Various types of capping agents have been used to encapsulate QDs into ZIF-8 nanopores. An effective method for encapsulating CdS QDs into ZIF-8 is to use 2-mercaptoimidazole as the capping agent. This is because 2-mercaptoimidazole is similar to the imidazolate ligands of ZIFs and can used for capping active species with simultaneous encapsulation during the crystal growth of ZIF-8. Compared to other widely used capping agents such as polyvinylpyrrolidone (PVP), using 2-mercaptoimidazole for encapsulating CdS QDs into ZIF-8 revealed photocatalytic effects along with the molecular sieving effect when using differently sized molecular redox mediators such as methyl viologen (MV^2+^) and diquat (DQ^2+^).

## 1. Introduction

Metal sulfide quantum dots (QDs) demonstrate tremendous potential for application in photocatalytic fields because of their high optical quantum yield, photostability, and high photocatalytic performance [1,2,3,4,5]. However, QDs show a strong tendency to aggregate into larger particles due to their high surface energy, leading to the loss of their unique characteristics. This tendency obstructs the utilization of QDs in photocatalytic applications [6,7,8]. To address this issue, several studies have explored the concept of the encapsulation of QDs into high porous scaffolds such as mesoporous materials [9,10], zeolites [11,12], polyoxometalate [13,14], and metal organic frameworks (MOFs) [15,16,17,18,19,20,21,22,23,24,25]. Recently, zeolitic imidazole frameworks (ZIFs), which are a class of MOFs, have been considered as an attractive class of porous scaffold materials for the incorporation of various components into the ZIF-8 network, owing to their high tunability and chemical and moisture stability compared to other MOFs [26,27,28]. Therefore, encapsulating functional QDs as catalytic sites into highly porous ZIF materials is a rational approach. QDs@ZIF composites were fabricated for inducing subsequent ZIF heterogenous growth around QDs to anchor an appropriate binder molecule on the surface. The most significant advantage of QDs@ZIF composites is the ability to customize the composition, size, and morphology of QDs for specific purposes, with the chemical properties of QDs retained after encapsulation within the crystalline hosts.

Lu et al. reported an exquisite approach in which a variety of QDs were encapsulated into ZIF-8 by employing polyvinylpyrrolidone (PVP) as the surfactant for surface modification of the QDs [15]. Several studies have reported this strategy of encapsulating QDs, including PVP-covered QDs such as CdS, CdTe, and CdSe, or nanoparticles, into ZIF-8 growth to be very effective [16,17,18,19,20]. In particular, the widely used method employing PVP as the binder between QDs and ZIF-8 is limited to complete removal of PVP after growth. This may reduce the photocatalytic activity of embedded QDs with respect to the accessibility of the catalytic sites. Despite the good dispersion of QDs in ZIFs, the possibility of the presence of unencapsulated QDs on the ZIFs’ surface remains due to the different growth rates of QD-Zn^2+^ and ligand-Zn^2+^. This interferes with the molecular sieving effect, which is one of the significant advantages in catalytic application.

**Scheme 1:**
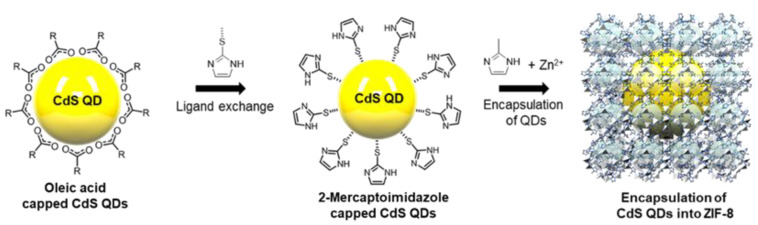
Schematic illustration of the ligand exchange process of oleic acid with 2-mercapto imidazole and encapsulation of exchanged CdS QDs into ZIF-8.

To adjust the growth rates for encapsulating active species into ZIFs, an effective alternative is to use agents similar to the imidazolate ligands of ZIFs used for capping active species. Therefore, 2-mercaptoimidazole could be a suitable capping material for realizing the above strategy to encapsulate nanoparticles inside ZIF-8. Herein, we report an effective strategy for the in situ encapsulation of CdS QDs as a representative metal sulfide by employing parallel capping agent 2-mercaptoimidazole with ligand-ZIF-8 into nanocages of ZIF-8 for various loading amounts of CdS QDs, as illustrated in Scheme 1. We also reveal the complete molecular sieving effect of the CdS QD@ZIF-8 composite prepared using the proposed strategy.

## 2. Materials and Methods

### 2.1. Materials

Cadmium oxide (98.5%, Sigma-Aldrich, St. Louis, MO, USA), oleic acid (extra pure, Sigma-Aldrich, St. Louis, MO, USA), 1-octadecene (90%, Acros, NJ, USA), sulfur powder (99.5%, Alfa, Ward Hill, MA, USA), chloromethane (extra pure, DUKSAN, Daejeon, Republic of Korea), polyvinylpyrrolidone (average M.W.: 58,000, Alfa, Ward Hill, MA, USA), 2-mercaptoimidazole (98%, %, Alfa, Ward Hill, MA, USA), 3-mercaptopropionic acid (99%, Alfa, Ward Hill, MA, USA), tetramethylammonium hydroxide (25% *w/w* in methanol, %, Alfa, Ward Hill, MA, USA), zinc nitrate hexahydrate (99%, Alfa, Ward Hill, MA, USA), cobalt nitrate (97%, Junsei, Tokyo, Japan), 2-methylimidazole (99%, Acros, NJ, USA), methyl viologen hydrate (98%, Acros, NJ, USA), diquat dibromide monohydrate (95%, Sigma-Aldrich, St. Louis, MO, USA), ammonium hexafluorophosphate (99%, Acros), triethanolamine (99%, Acros, NJ, USA)

### 2.2. Synthesis of Oleic Acid Capped CdS QDs

CdS QDs were prepared using the hot-injection method according to previously reported methods [29,30,31]. To obtain the cadmium source, 1-octadecene (ODE) (36 mL), CdO (3.0 mmol), and oleic acid (5 mL) were mixed in a two-neck round-bottom flask and dissolved by heating to 280 °C under an argon atmosphere. Separately, as the sulfur source, sulfur powder (1.5 mmol) was poured into an ODE (5 mL) solution in a round-bottom flask and completely dissolved by heating to 80 °C. The sulfur solution was swiftly injected into the prepared hot Cd source solution. The mixture was cooled to 220 °C and maintained at this temperature for 1 h to facilitate the growth of CdS QDs. After the mixture was cooled to room temperature, the synthesized CdS QDs were centrifuged several times for removing unreacted chemicals with 1:1 acetone/ethanol mixture (1:1). The pure CdS QDs capped with oleic acid were dissolved in chloroform.

### 2.3. Preparation of 2-Mercaptoimidazole Capped CdS

Surface ligand exchange of CdS QDs with thiol-based ligands such as 2-mercaptoimidazole(2-MIm) was conducted according to the literature [31]. First, 2-MIm (1 mL) was dispersed in 1:1 chloroform/methanol (20 mL) and the pH value was adjusted to approximately 11 using a methanolic tetramethylammonium hydroxide (TMAOH) solution. The synthesized QDs (30 mg) were added to this mixture and stirred at 50 °C for 12 h in the dark. After the exchange, the CdS QD products were washed using centrifugation with an acetone/ethanol mixture (1:1).

### 2.4. Encapsulation of CdS QDs into ZIF-8

ZIF-8 crystals were synthesized according to previous reports [16,17,18,19,20]. For ZIF-8, first, 0.9 g (3.0 mmol) of Zn(NO_3_)_2_∙6H_2_O was dissolved in 30 mL of MeOH. Separately, 0.984 g (12.0 mmol) of 2-methylimidazole was completely dissolved in 10 mL of MeOH and the desired amount of methanol solution of ligand-exchanged CdS QDs was added. Zn solution was added into a mixture of 2-methylimidazole and ligand-exchanged CdS QDs; the mixture was stirred for 12 h at room temperature. After pale yellow CdS QD@ZIF-8 crystals were formed, the solution was centrifuged at 8000 rpm for 10 min to facilitate separation, and the crystals were washed with methanol five times.

### 2.5. Test for Photocatalytic Performance

Firstly, we prepared two molecular redox mediators such as methyl viologen (denoted as MV^2+^) and diquat (denoted as DQ^2+^) with anion PF^6−^. Methyl viologen dichloride and diquat dibromide with the anions PF^6−^ are soluble in acetonitrile. Anion exchange was conducted to convert halide to PF^6−^ by stirring the methyl viologen dichloride (0.1 g) and diquat dibromide (0.1 g) solution in the presence of ammonium hexafluorophosphate (0.3 g). To investigate the photocatalytic performance of CdS-QD@ZIF-8, in situ measurements of the spectral change from MV^2+^ to MV^+^ in the presence of CdS-QD@ZIF-8 were carried out as follows. CdS-QD@ZIF-8 (2.0 mg) was added in a photoreaction cell containing 4 mL of 0.2 mM acetonitrile solution of MV^2+^ and 1.0 mM of triethanolamine (TEAOH), and the cell was bubbled with high-purity argon for 3 min and air-tightened lid under the dark. The airtight fluorescence cell was irradiated with a blue LED lamp (415 nm, 1 W) at room temperature for the desired time.

### 2.6. Instrumentation

XRD patterns of the samples were obtained using an X’Pert-MPD System (PHILIPS, Eindhoven, Netherlands) diffractometer with monochromatic Cu Kα radiation in the 2*θ* ranges from 10° to 60° at 40 kV and 40 mA. Scanning electron microscopy (SEM) images were obtained using a CX-200 scanning electron microscope (COXEM, Daejeon. Republic of Korea). Transmission electron microscopy (TEM) analysis was conducted using a JEM-2100F microscope (JEOL, Tokyo, Japan) operated at 200 kV. Infrared spectra were recorded on a FT/IR-4000 (JASCO, Tokyo, Japan) system in the spectra range from 400 to 4000 cm^−1^. All UV–vis absorption spectra were obtained using on a UV-2600 UV–vis spectrophotometer (Shimadzu, Kyoto, Japan). For determining the amount of encapsulated CdS into ZIF-8, we obtained the values of Cd^2+^ to Zn^2+^ ratio of the series of CdS@ZIF-8 samples using inductively coupled plasma–optical emission spectrometry (ICP-OES, Optima 7300 DV, PerkinElmer, Waltham, MA, USA). Nitrogen adsorption–desorption isotherms of the series of CdS QDs@ZIF-8 composites for evaluating microporous characteristics were obtained from a BEL sorp-Max instrument (BEL, Tokyo, Japan). Before the isotherms were measured, all of the samples were pretreated by evacuation (∼20 mTorr) at 150 °C for 12 h.

## 3. Results

### 3.1. Characterization of ZIF-8 Encapsulated CdS QDs

**Figure 1:**
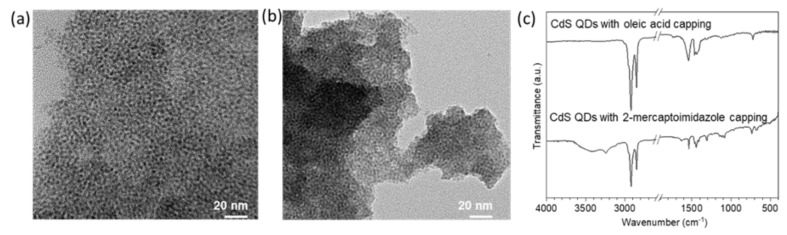
TEM images of CdS QDs capped with (**a**) oleic acid, (**b**) 2-mercaptoimidazole, and (**c**) infrared spectra as indicated.

We synthesized CdS QDs with sizes in the range of 3–5 nm in the presence of oleic acid as a surface capping ligand to avoid aggregation. Morphological characterization of the pristine CdS QDs was carried out using transmission electron microscopy (TEM). From Figure 1a, a fairly uniform size distribution of nanoparticles is observed, with an estimated mean size centered at 3–4 nm. Even after ligand exchange with 2-MIm, the ligand exchange process did not cause any structural and morphological changes in CdS QDs, except the partial aggregation of QDs, as seen Figure 1b. This result is consistent with identical first exitonic absorption obtained for pristine and 2-mIm exchanged CdS QDs. In addition, FTIR spectroscopy confirmed the success of ligand exchange (Figure 1c). As seen, the IR spectrum of the CdS quantum dot exchanged with 2-MIm showed the characteristic band at 1610 and 3252 cm^−1^ corresponding to the –C–N stretching and N–H stretching frequency of the imidazole ring, respectively.

**Figure 2:**
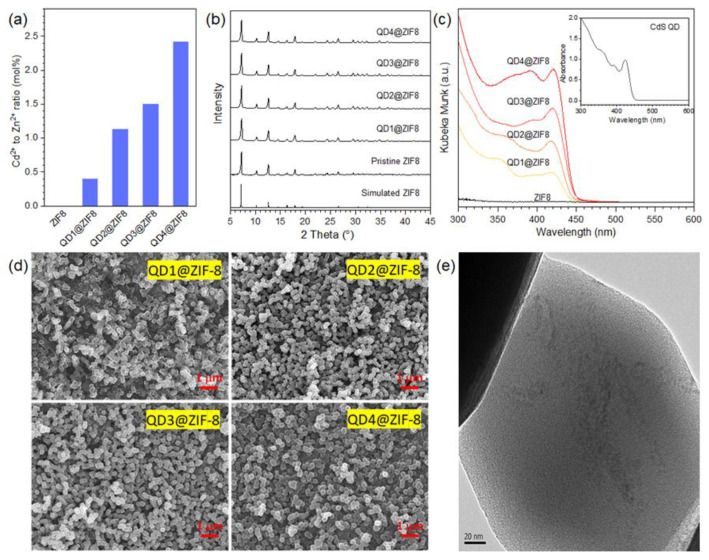
(**a**) Cd^2+^ to Zn^2+^ ratio from ICP-OES analysis; (**b**) XRD patterns; (**c**) UV–vis diffuse reflectance spectra represented as Kubelka–Munk function; (**d**) SEM images of CdS QDs@ZIF-8 and (**e**) representative TEM image of CdS QD3@ZIF-8.

We encapsulated different amounts of ligand-exchanged CdS QDs in ZIF-8 under identical synthetic conditions as in the case of pristine ZIF-8 by simply adjusting the amount of CdS QD solution to be added. To verify the amount of CdS QDs encapsulated into ZIF-8, the mole percent of Cd^2+^ per mole of Zn^2+^ in the series of QDs@ZIF-8 samples was determined using ICP-OES (Figure 2a). We denoted each series of QDs@ZIF-8 as QD1@ZIF-8, QD2@ZIF-8, QD3@ZIF-8, and QD4@ZIF-8, corresponding to ratios of 0.4, 1.1, 1.6, and 2.4, respectively. To evaluate the successive encapsulation CdS QDs into ZIF-8 without structural change, the prepared QDs@ZIF-8 at various loading amounts was analyzed by XRD, as seen in Figure 2b. The characteristic peaks of the ZIF-8 structure for the series of QDs@ZIF-8 appeared at 7.3°, 10.3°, 12.6°, 14.6°, 16.3°, and 17.9°, corresponding to (0 1 1), (0 0 2), (1 1 2), (0 2 2), (0 1 3), and (2 2 2) planes, respectively. These peaks were sharp and their intensity ratios were consistent with the simulated and measured XRD pattern for pristine ZIF-8 [20,26]. This indicates that the ZIF-8 framework was well preserved after the encapsulation of CdS QDs during crystallization. The diffuse reflectance UV–visible (UV–vis) spectra of the series of QDs@ZIF-8 composites with different amounts of encapsulated CdS QDs are compared in Figure 2c. First, the exitonic absorption band at 420 nm by encapsulated CdS QDs was almost identical to that of pristine CdS QDs (inset of Figure 2c) and increased in intensity with increasing amount of CdS QDs. The identification absorption bands represent sustenance of 2-MIm capped CdS QDs after the encapsulation process. The FE-SEM images of the obtained series of QDs@ZIF-8 indicate that their morphology and size of around 0.2 μm were almost identical. This indicates that the encapsulation of 2-mercaptoimidazole ligand-exchanged QDs does not affect the growth of ZIF-8 during crystallization because of the isostructure formed between the QD capping ligand and the ZIF-8 ligand. The TEM image of QDs@ZIF-8 clearly shows that the encapsulated CdS QDs are moderately monodispersed on the outer surface of the ZIF-8 crystals, and all CdS QDs are clearly encapsulated into the ZIF-8 matrix (Figure 2e). The nitrogen adsorption and desorption isotherms of CdS QDs@ZIF-8 and pristine ZIF-8 were obtained for clarifying microporous natures in Figure S1a. As seen, although the amount of nitrogen adsorption is found to be slightly lower in CdS QDs@ZIF-8 than pristine ZIF-8 due to the increasing amount of the loading of CdS quantum dots, all samples exhibit type I isotherm, implying the microporous characteristics. Furthermore, from the size distribution plot, the encapsulation of CdS QDs does not alter the pore size distribution of ZIF-8, as seen in Figure S1b. 

### 3.2. Photocatalytic Performance of ZIF-8 Encapsulated CdS QDs

**Scheme 2:**
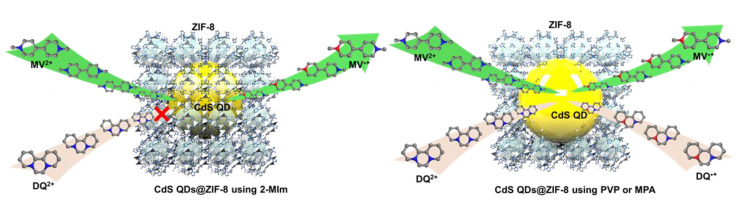
Schematic illustration of molecular sieving effect of CdS QD@ZIF-8 for MV^2+^ and DQ^2+^ photoreaction depending on capping agents as indicated.

**Figure 3:**
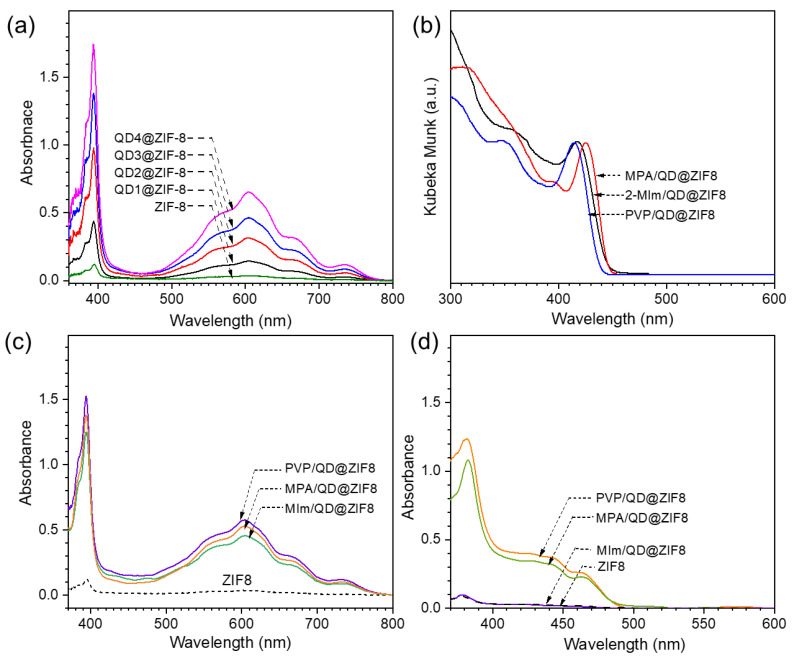
(**a**) UV–vis difference spectra for photoreduction of MV^2+^ using CdS QD@ZIF-8 composites with different CdS QD loadings; (**b**) UV–vis diffuse reflectance spectra represented as the Kubelka–Munk function for CdS QD@ZIF-8 with different capping agents, and UV–vis difference spectra for photoreduction of (**c**) MV^2+^ and (**d**) DQ^2+^ using CdS QD@ZIF-8 with different capping agents.

MV^2+^ and DQ^2+^ of different sizes are widely investigated molecular redox mediators in the CdS photocatalyst system. We hypothesized that MV^2+^ can access the CdS QDs encapsulated ZIF-8 via mass transfer through the window but DQ^2+^ cannot, as shown in Scheme 2. To prove the molecular sieving effect of QDs@ZIF-8, we tested the photoreduction of MV^2+^ and DQ^2+^ using QDs@ZIF-8 composites as the visible photocatalyst and triethanolamine (TEOA) as a sacrificial electron donor in acetonitrile. When the QDs@ZIF-8 composite was irradiated with blue LED (415 nm, 1 W), the solution turned blue and the color intensified with time as irradiation continued. The UV–vis spectra of the blue supernatant solutions from the QDs@ZIF-8 suspensions showed the two characteristic absorption bands at 397 and 605 nm of the viologen radical cation, MV^•+^, and their intensity increased with photoreaction duration, as shown Figure 3a. In the control experiment in the dark, no absorption band of MV^•+^ was detected. For a more systematic study, we conducted photoreduction using the QDs@ZIF-8 composites with different loading amounts of CdS QDs including pristine ZIF-8. Apparently, as the loading amount increased, photoinduced reduction of MV^2+^ effectively occurred, as seen in Figure 3a. This indicated that ZIF-8 itself does not thermally reduce MV^2+^ to MV^•+^ and that the photoinduced reduction of MV^2+^ originates from encapsulated CdS QDs. Using identical photoreaction conditions, the photoactivity of CdS QDs@ZIF-8 composites using other types of binding agents such as mercaptopropionic acid (MPA) and PVP, which exhibited a CdS QD absorption band as described in Figure 3b, were measured and compared with that obtained using mIm. All the samples displayed almost identical performance in the case of MV^2+^ (Figure 3c). In the presence of bulkier DQ^2+^ (compared to the window of ZIF-8), CdS QDs@ZIF-8 using 2-MIm did not photoproduce DQ^•+^. Furthermore, when several different binding molecules such as PVP and MPA, which are commonly used to encapsulate QDs into ZIF, were employed, CdS QDs@ZIF-8 photoproduced DQ^•+^ by the external effect of CdS QDs on the ZIF-8, as seen in Figure 3d. Due to the steric hinderance, compared with pyridinium parts at the end of para position of MV^2+^, pyridinium parts at the ortho position of DQ^2+^ cannot contact encapsulated CdS quantum dots, resulting in absolute regioselectivity. This indicated that the molecular sieving effect of CdS QDs@ZIF-8 using 2-MIm was similar to that of the ligand-ZIF-8. This result reveals that the utilization of identical binding molecules is a rational approach for generating QD@ZIF composites.

## 4. Conclusions

We developed a feasible method for the encapsulation of CdS QDs into ZIF-8. The CdS QDs capped with 2-mercaptoimidazole could be readily encapsulated, with simultaneous growth of the ZIF-8 crystals. Compared to other widely used capping agents such as PVA, using 2-mercaptoimidazole for encapsulating CdS QDs into ZIF-8 revealed the excellent photocatalytic performance of the QDs@ZIF composites. Moreover, outstanding molecular sieving effects with differently shaped molecular redox mediators such as MV^2+^ and DQ^2+^ were achieved, owing to the effective encapsulation of the CdS QDs. This strategy can be readily applied to other types of ZIFs for extending the photocatalytic application of composites fabricated from metal chalcogenide QDs and ZIFs.

## Supplementary Materials

The following are available online at https://www.mdpi.com/2079-4991/10/12/2498/s1, Figure S1: (a) Nitrogen adsorption (solid) and desorption (cycle) curves and (b) pore size distribution of CdS QDs@ZIF-8 samples.
