# Supplementary material for: Strategy for Encapsulation of CdS Quantum Dots into Zeolitic Imidazole Frameworks for Photocatalytic Activity
Source: Nanomaterials (Basel). 2020 Dec 12;10(12):2498. doi: 10.3390/nano10122498 (PMC7764424; doi:10.3390/nano10122498)
Supplement: Supplementary file 1 [file nanomaterials-10-02498-s001.pdf]

nanomaterials

Supplementary Materials

Strategy for Encapsulation of CdS Quantum Dots into Zeolitic Imidazole Frameworks for Photocatalytic Activity

Ye Rim Son, Minseok Kwak, Songyi Lee and Hyun Sung Kim

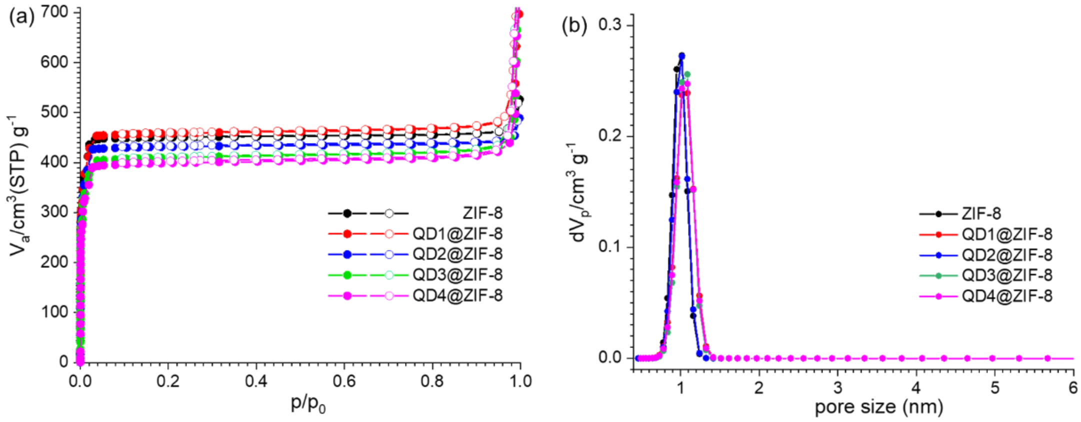

Figure S1. (**a**) Nitrogen adsorption (solid) and desorption (cycle) curves and (**b**) pore size distribution of CdS QDs@ZIF-8 samples.
